# Myocardial Infarction in a Patient With Hereditary Hemorrhagic Telangiectasia: A Case Report and Review of Literature

**DOI:** 10.7759/cureus.15219

**Published:** 2021-05-24

**Authors:** Suman Rao, Alisha Khan, Dana Aiello

**Affiliations:** 1 Internal Medicine, State University of New York Upstate Medical University, Syracuse, USA; 2 Cardiology, State University of New York Upstate Medical University, Syracuse, USA

**Keywords:** hereditary hemorrhagic telangiectasis, st-elevation myocardial infarction (stemi), osler-weber-rendu syndrome, osler-weber-rendu, cardiac catheterization

## Abstract

Hereditary hemorrhagic telangiectasia (HHT) is an autosomal dominant disorder that results in vascular defects and arteriovenous malformations. We present a 25-year-old male with a past medical history of HHT who came in with chest pain and was found to have an ST-elevation myocardial infarction (STEMI) and subsequently received a bare-metal stent to the mid-left anterior descending artery (LAD). Although there is a predisposition for bleeding, HHT can lead to thrombotic manifestations such as myocardial infarction (MI), as seen in our patient. Healthcare providers should be aware of this association to be able to efficiently diagnose acute coronary syndrome in HHT patients. Further studies are required to assess the efficacy of bare-metal stents in HHT patients who present with an MI.

## Introduction

Hereditary hemorrhagic telangiectasia (HHT), also known as Osler-Weber-Rendu syndrome, is an autosomal dominant disorder that results in vascular defects, such as arteriovenous malformations and telangiectasias [1]. Although most complications of this syndrome consist of epistaxis, gastrointestinal bleeding, and cerebrovascular hemorrhage, there are several instances where HHT can cause thrombotic/embolic effects, which could result in myocardial infarction (MI), as was the case in our patient.

## Case presentation

We present a 25-year-old male with a past medical history of HHT, ischemic cerebrovascular accident with residual right hemiparesis, left parietal arteriovenous malformation for which he underwent coiling, and localization-related epilepsy who presented to the ED with complaints of nausea, vomiting, abdominal pain, and chest pain that had been going on for the past five days. His only home medication was valproic acid.

Vital signs consisted of blood pressure of 113/72 mmHg, heart rate of 98 bpm, respiratory rate of 22 bpm, oxygen saturation of 93% on room air. Physical examination revealed an obese male with a BMI of 33.9 kg/m2 in moderate cardiopulmonary distress. On auscultation, normal heart sounds and vesicular breath sounds were heard. Our patient had right-sided hemiparesis with 2/5 muscle strength in the upper and lower extremities.

**Figure 1:**
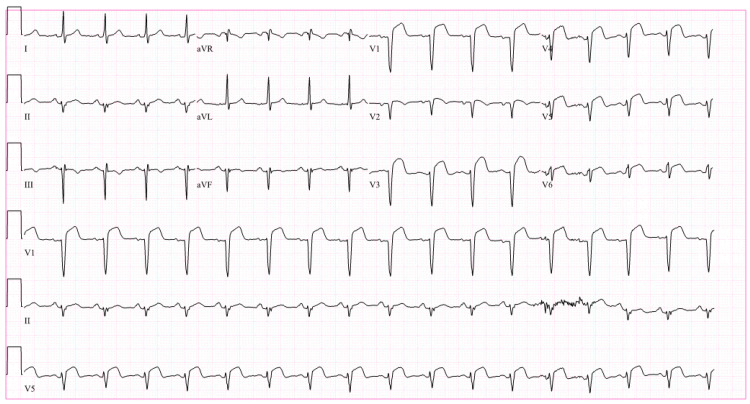
Electrocardiography. Electrocardiography (EKG) was done which showed a ventricular rate of 87 bpm, PR interval of 140 milliseconds, QRS duration of 86 milliseconds, a QTC of 397 milliseconds, a sinus rhythm with left axis deviation, and ST-segment elevations in the anterolateral leads.

Lab work revealed potassium of 3.6 mEq/L, creatinine of 1.3 mg/dL, and a glucose of 92 mg/dL. Troponin T was noted to be elevated at 2.27 ng/mL. Lipid profile showed a cholesterol of 137 mg/dL, high-density lipoprotein (HDL) cholesterol of 24 mg/dL, low-density lipoprotein (LDL) cholesterol of 91 mg/dL, triglycerides of 115 mg/dL, and very-low-density lipoprotein (VLDL) cholesterol of 23 mg/dL. Electrocardiography (EKG) was done which showed a ventricular rate of 87 bpm, PR interval of 140 milliseconds, QRS duration of 86 milliseconds, a QTC of 397 milliseconds, a sinus rhythm with left axis deviation, and ST-segment elevations in the anterolateral leads (Figure 1).

An urgent cardiac catheterization was pursued which showed a 90% thrombotic occlusion in the mid-left anterior descending (LAD) artery, and overlapping bare-metal stents were placed after percutaneous coronary intervention. Bare-metal stents were chosen due to the risk of bleeding secondary to a history of HHT. The right coronary artery (RCA) and left circumflex were noted to be normal. Left ventricular end-diastolic pressure (LVEDP) was measured at 30 mmHg.

**Figure 2:**
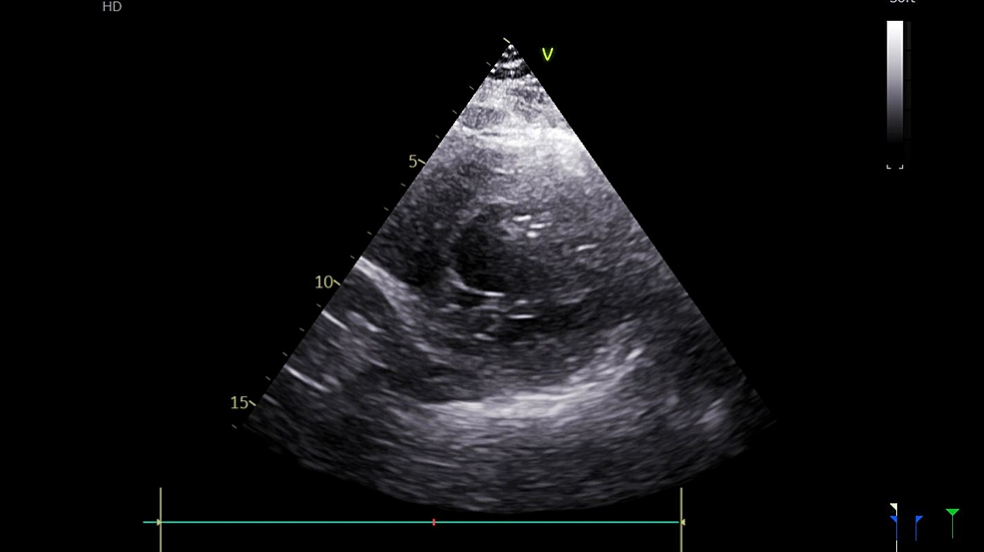
Echocardiogram in the diastolic phase.

**Figure 3:**
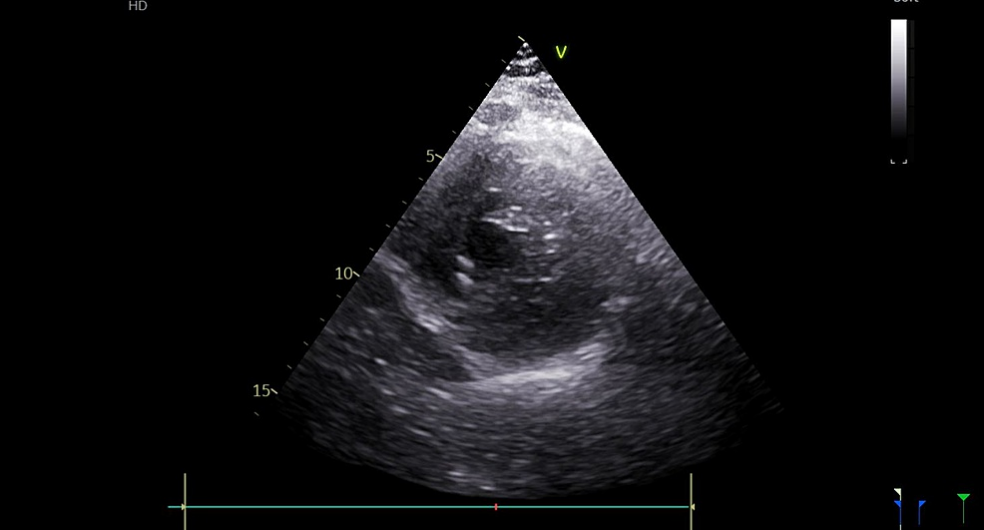
Echocardiogram in the systolic phase. Post-procedure transthoracic echocardiogram (TTE) showed a left ventricular ejection fraction (LVEF) of 44%, and hypokinesis of the mid to distal septum and apex.

Post-procedure transthoracic echocardiogram (TTE) showed a left ventricular ejection fraction (LVEF) of 44%, and hypokinesis of the mid to distal septum and apex (Figures 2 and 3). Troponin was noted to downtrend to 1.9 ng/mL and subsequently to 1.61 ng/mL. He was started on aspirin 81 mg, clopidogrel 75 mg, atorvastatin 80 mg daily, and metoprolol tartrate 25 mg twice daily. He was counseled to continue dual antiplatelet therapy for one month and then continue on the aspirin alone.

Our patient was observed in the cardiac ICU unit for 48 hours post catheterization and was then discharged on the medication therapy described above. He was able to be followed up outpatient and was compliant with his one month of clopidogrel. He did not complain of any bleeding events, during this time.

## Discussion

HHT, previously referred to as Osler-Weber-Rendu syndrome, is an autosomal dominant disorder that is characterized by the manifestation of several vascular lesions including arteriovenous malformations and telangiectasias. Defects in ENG, ACVRL1, and SMAD4 genes are implicated in the pathogenesis of this disease, as they are thought to cause vascular deformities [2].

Generally, HHT patients present with mucocutaneous telangiectasias that result in epistaxis, gastrointestinal bleeding, iron deficiency anemia, cerebrovascular manifestations (such as hemorrhagic strokes), or high-output cardiac failure [3]. The diagnosis of this disease is largely clinical with the diagnostic criteria being spontaneous episodes of epistaxis, multiple sites of telangiectasia, visceral vascular abnormalities, and/or a first-degree relative with HHT. Genetic testing can also be pursued [2]. Although HHT patients are largely tied with hemorrhagic and bleeding events, it should be noted that HHT is also associated with thrombotic events [4]. There are cases where HHT patients have presented with pulmonary embolism and Budd-Chiari syndrome [5].

In addition, there are several cases that propose that pulmonary arteriovenous malformations (PAVM) lead to paradoxical embolism, due to the presence of a right to left shunt. The study performed by Clark et al. suggests that patients with untreated PAVM were at greater risk of MI secondary to a paradoxical embolism [6]. Martínez-Quintana et al. discuss a 46-year-old woman with HHT who presented with MI secondary to the presence of PAVM [7]. PAVM would be visualized by a CT pulmonary angiogram, which was not pursued in our patient. 

Dospinescu et al. discuss the lack of data available in the treatment of STEMI in a patient with HHT. They state that the tPA contraindication in a patient with HHT led to the initiation of a heparin drip, and an ensuing delay in care with transport to a catheterization center [8]. Our case demonstrates the importance of being aware of MI in HHT patients, especially in a younger patient population. We decided to place a bare-metal stent due to the decreased length of antiplatelet therapy that would be required. Research to understand the etiology of thrombosis/embolization in developing MI in the HHT population is necessary. In addition, further studies are required to assess the efficacy of bare-metal stents as compared to drug-eluting stents in the HHT patient population who seem to carry both thrombotic and bleeding risks.

## Conclusions

Although there is a predilection for bleeding and hemorrhage, HHT can predispose patients to thrombotic/embolic manifestations such as MI, as seen in our patient. Healthcare providers should be aware of this association to be able to streamline the diagnostic workup of an HHT patient presenting with signs of acute coronary syndrome. Further studies are required to assess the utility of screening for PAVM with CT pulmonary angiography and subsequent embolization, as well as the efficacy of bare-metal stents in HHT patients who present with an MI.
